# Self and directed assembly: people and molecules

**DOI:** 10.3762/bjoc.12.42

**Published:** 2016-03-01

**Authors:** Tony D James

**Affiliations:** 1 Department of Chemistry, University of Bath, Bath, BA2 7AY UK

**Keywords:** boronic acids, fluorescence, glucose sensor, self and directed assembly, supramolecular

## Abstract

Self-assembly and directed-assembly are two very important aspects of supramolecular chemistry. As a young postgraduate student working in Canada with Tom Fyles my introduction to Supramolecular Chemistry was through the self-assembly of phospholipid membranes to form vesicles for which we were developing unimolecular and self-assembling transporter molecules. The next stage of my development as a scientist was in Japan with Seiji Shinkai where in a “Eureka” moment, the boronic acid templating unit (directed-assembly) of Wulff was combined with photoinduced electron transfer systems pioneered by De Silva. The result was a turn-on fluorescence sensor for saccharides; this simple result has continued to fuel my research to the present day. Throughout my career as well as assembling molecules, I have enjoyed bringing together researchers in order to develop collaborative networks. This is where molecules meet people resulting in assemblies worth more than the individual “molecule” or “researcher”. My role in developing networks with Japan was rewarded by the award of a Daiwa-Adrian Prize in 2013 and I was recently rewarded for developing networks with China with an Inaugural CASE Prize in 2015.

## Review

### Beginnings

When at school I became distracted very easily and while this may not be a good thing for getting on in education it is the one aspect of my personality I have found to help me as a scientist. Since being easily bored can drive you to look for something more interesting – in science the something more interesting is a new discovery. Therefore, while getting side-tracked is not a very good thing in everyday life, for me it has allowed me to flourish as a scientist, since being “side-tracked” often opens up new and exciting areas of research not possible if you just follow the flock.

At school and as teenager in the early 1980’s I needed to decide what to do as a career. Luckily, around this time of decision in my life I happened to watch a programme by the Nobel Prize Physicist Richard Feynman on Horizon in 1981 [1] “The Pleasure of Finding Things Out”. His eloquence and ability to convey science to everyone amazed me and inspired me to want to experience the same joy in science that he had in abundance.

Richard P. Feynman (1918–1988) explains how he has an artist friend who describes a flower he is holding *“I as an artist can see how beautiful this is but you as a scientist take this all apart and it becomes a dull thing,”* Richard P. Feymann does not agree, since as a scientist he can observe the beautiful inner workings and structure of the flower which adds new layers and dimension to the observation of the flower*.*

Interestingly at this time I was probably a better artist than scientist, and so this comment by Feynman struck a very strong cord with me. Therefore, in 1984 after leaving the Abraham Darby School in Telford, I chose to go to The University of East Anglia (UEA) to study chemistry. So why chemistry? To be truthful this is a combination of factors. Firstly, I found chemistry to be the most interesting and exciting science in school. Secondly, the chemistry industry in the UK was very strong, thus, on finishing my degree I anticipated that a suitable job would be available (an important factor given my working class roots). Thirdly, the chemistry course at UEA offered a one year placement in the USA [2]. While these types of courses are now very common at the time this was a novel concept.

Therefore, I spent a year (1984-1985) as a visiting student at the University of Massachusetts in Amherst. The year I spent at UMass (ZooMass) contributed to my love of American recreational culture and travelling in general. The year in Amherst started the chain reaction of events leading to my current career. This starts with my move in 1986 to Canada and the University of Victoria for a PhD with Tom Fyles and is followed by a move in 1991 to Kurume, Japan to work with Seiji Shinkai as a Postdoctoral Research Fellow. Finally, in 1996 after 10 years outside the UK, I returned home to the University of Birmingham as a Royal Society University Research Fellow.

### Research

**Scheme 1:**
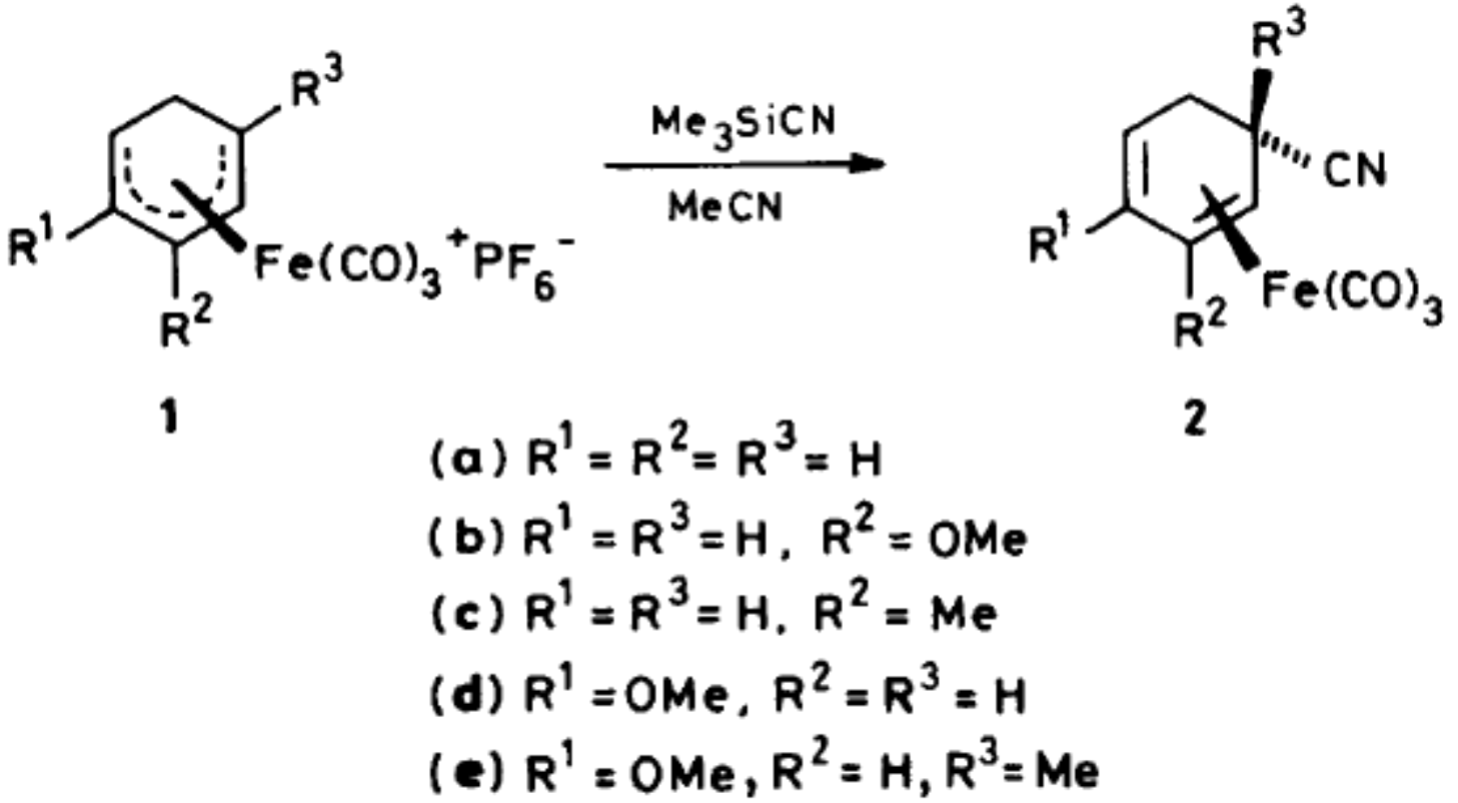
Reaction of trimethylsilyl cyanide with tricarbonyl (*η*5-cyclohexadienyl)iron(1+) salts. Reproduced with permission from [3]. Copyright 1987 Royal Society of Chemistry.

My first true research was at UEA and as a final year project student with G. Richard Stephenson [3]. This project opened my eyes to “research” as something you did rather than just read about. The project involved evaluation of the alkylation reaction of trimethylsilyl cyanide with tricarbonyl(*η*5-cyclohexadienyl)iron(1+) salts (Scheme 1). The reaction was shown to involve the isocyanide isomer, produced in rate-limiting pre-equilibration. The rate of reaction was proportional to the concentration of MeSiCN, with reactive metal complexes, but was independent of the concentration of the dienyl salt. With deactivated 2-methoxy substituted dienyl salts, a change in the rate-limiting step was observed.

Coupling my new found love of North America and research, I decided to look for a PhD and as it happens the University of Victoria had an advert in chemistry in Britain (Now Chemistry World) for PhD Fellowships in chemistry. The rest as they say is history, I applied was successful and started my research career in Victoria with Professor Thomas M. Fyles [4–10]. The choice of Tom as PhD mentor was a really great one for me. Tom is an outstanding scientist but more importantly for me he was a wonderful mentor. The project I worked on with Tom had many ups and downs and involved some quite tricky synthetic chemistry (which is not my forte – this will be attested to by all those who have worked with me and seen the pile of broken glassware in the wake of my synthetic efforts). However, throughout the project Tom was always very positive and full of new ideas when things did not quite work as we had hoped. It is through working with Tom that I realised that to do great science and make great discoveries was a roller coaster ride and he taught me a very important lesson which was that what we consider as “bad” results were just as important as the “good” ones. From working with Tom I learnt a lot and in particular how to be a good supervisor capable of nurturing (or at least knowing how to) good research – summing up in Tom’s own words this was the “take home message from my PhD.”

**Figure 1:**
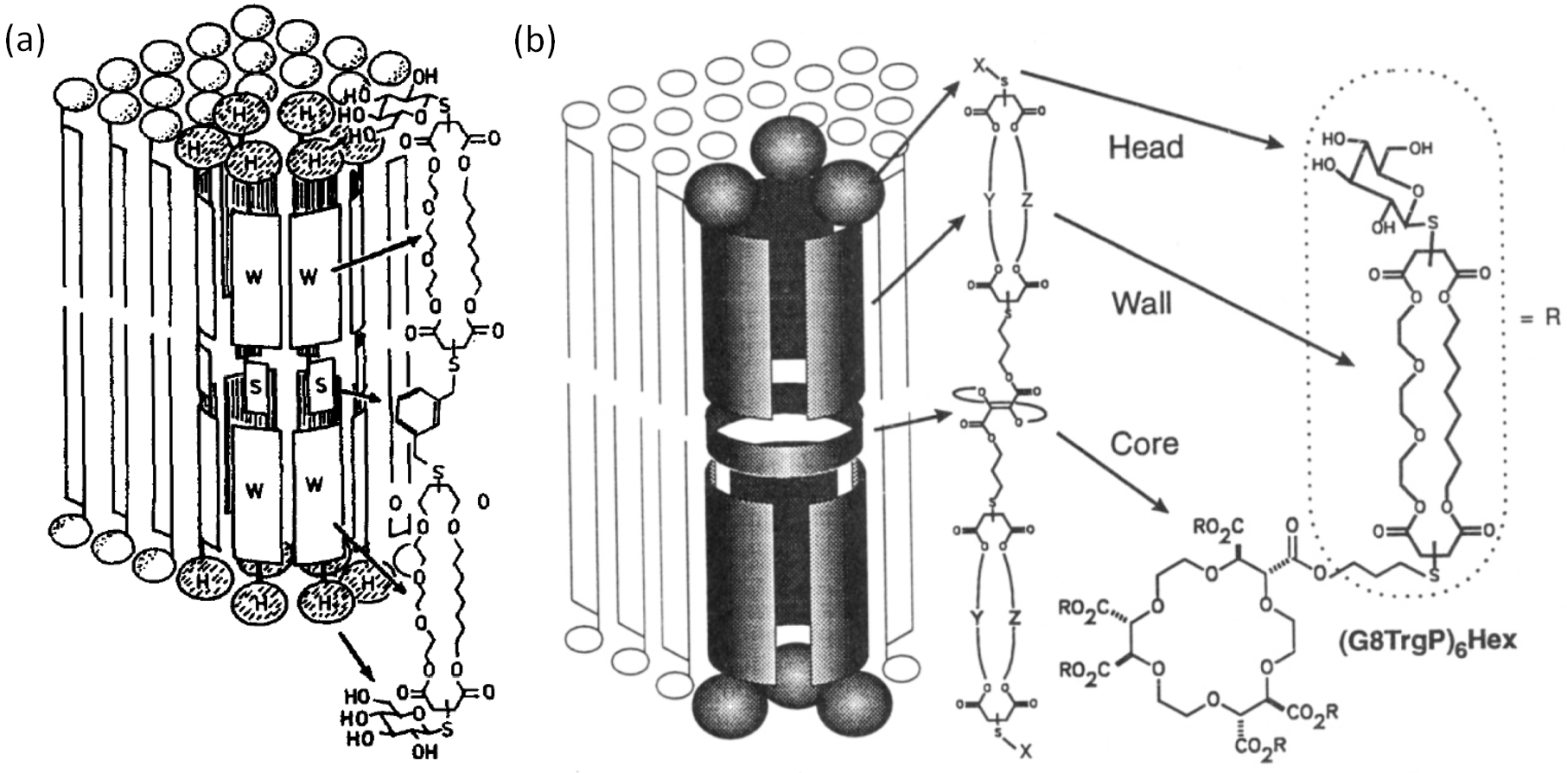
(a) Supramolecular pore formers. Reproduced with permission from [6]. Copyright 1990 Elsevier. (b) Unimolecular ion channel. Reproduced with permission from [9]. Copyright 1993 The American Chemical Society.

During my PhD we developed both self-assembling supramolecular pore formers as well as unimolecular ion channels for the transport of metal ions across biological membranes. The project required both the synthesis and evaluation of a number of transporter molecules (Figure 1).

#### Post-doctoral research

For many the PhD is enough so why did I want more? I think this is because Tom had shown me how to appreciate the bad results or research failures. I was also inspired by the tactile lecture of Donald J. Cram at Pacifichem (1987), where he sent a CPK model of a carcerand around the audience, and the 1990 lecture by Sir J. Fraser Stoddart in Halifax (Canada) where he used beautiful language and amazing colours to convey difficult concepts. (Note that at this time most lectures were colourless). Last but not least, I still had a strong desire to travel to a new research position in Japan as a research fellow for Seiji Shinkai as part of his newly formed ERATO (JST) Chemirecognics Project. This was quite a leap because other than for the fact that I was studying Karate at the University of Victoria, I knew very little about Japan and almost-nothing about the Japanese Language. (Karate had taught me to count from 1 to 10).

Having just finished a project with a lot of multistep synthesis, I was immediately drawn to projects as part of the boronic acid research group. The boronic acid group were developing carbohydrate receptors from what looked like simple molecules requiring just one or two steps to synthesize.

**Figure 2:**
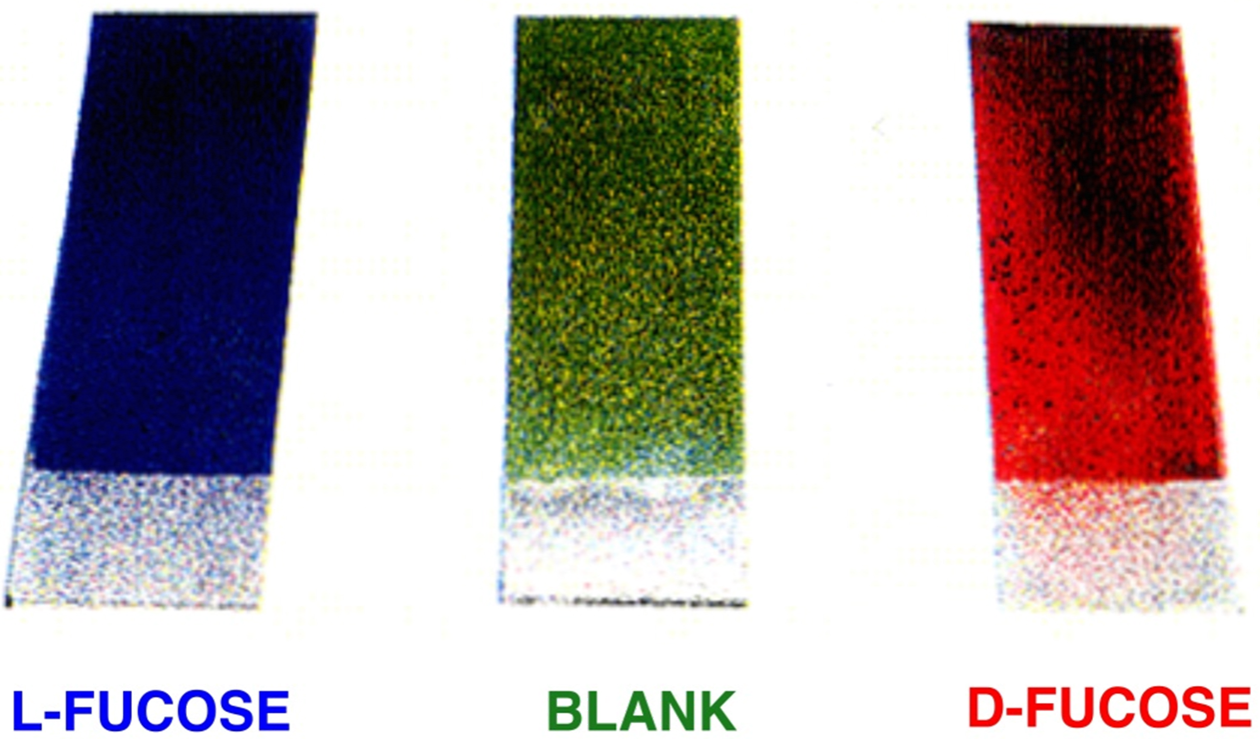
An intelligent liquid crystal to read out saccharide structure as a color-change. Picture provided by Seiji Shinkai, Director of the ERATO Chemirecognics Project (1990–95) [13].

Therefore, while working with the late Takaaki Harada, we developed a simple colorimetric system able to “read-out” the chirality of sugars. The system we developed used the colour of liquid crystals to determine chirality and was quickly prepared in just two steps. The addition of our D-glucose boronate complex to a liquid crystal system the colour changed from green to red, however for the L-glucose system the colour changed from green to blue [11–12] (Figure 2 depicting the same colour changes for D- and L-fucose). On observing these amazing colour changes of the “green” liquid crystal system [13], we realised that the obvious place to publish the work was “*Chem. Commun.*” [14] (given that the green was a similar colour of the cover [15] of the Journal at that time).

**Scheme 2:**
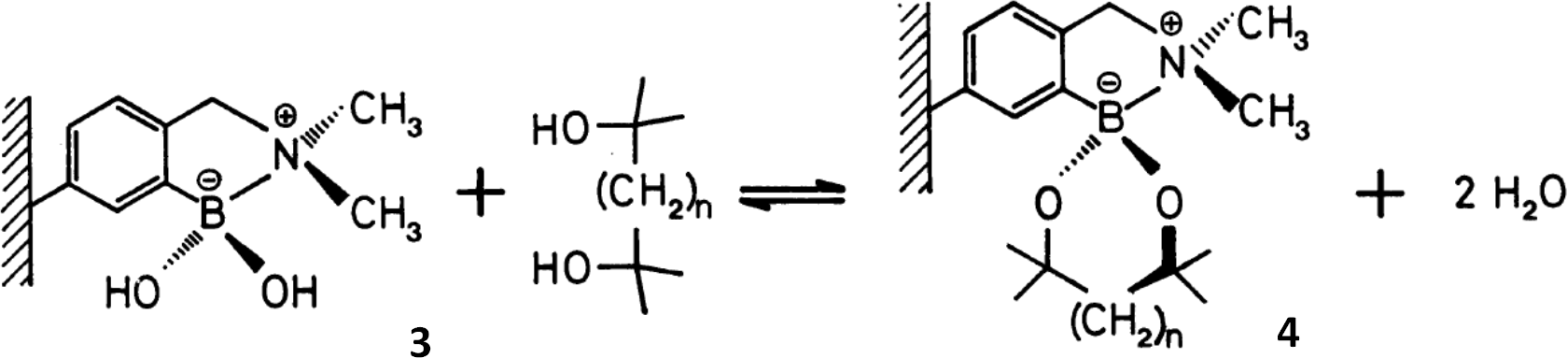
Polymeric boronic acid receptor units developed by Wulff. Reproduced from [16]. Copyright 1982 International Union of Pure and Applied Chemistry.

**Figure 3:**
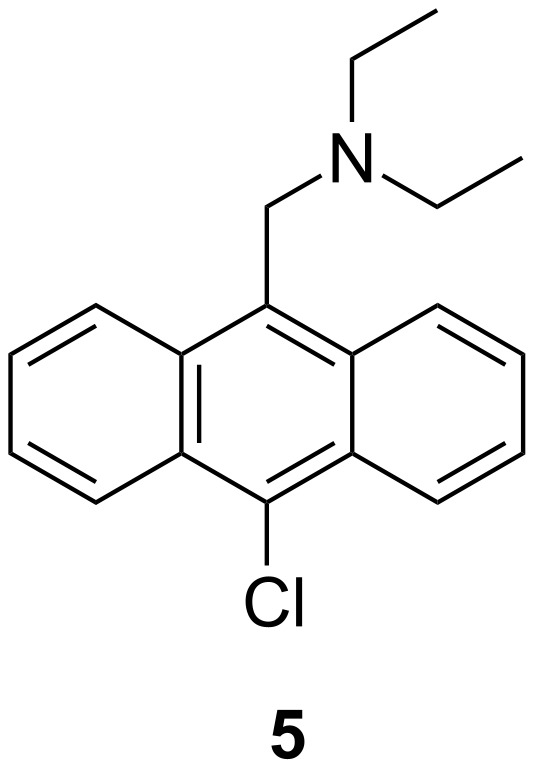
Fluorescence photoinduced electron transfer (PET) pH sensor developed by A. P. De Silva.

**Figure 4:**
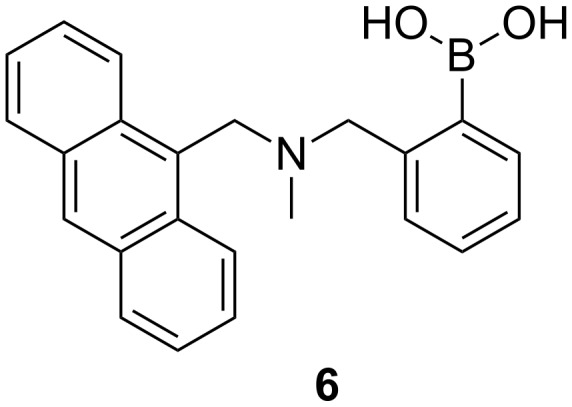
Fluorescence PET sensor for saccharides.

Following a truly inspiring talk by A. P. De Silva who visited the Chemirecognics project in Kurume and on a long flight back from a conference in the Netherlands (XVIII ISMC 1993, Enschede The Netherlands), I had the “good idea” to combine receptor units used by Gunter Wulff (Scheme 2) [16] and the fluorescent photoinduced electron transfer (PET) pH sensors developed by A. P. De Silva (Figure 3) [17] in order to develop a fluorescence sensor for saccharides [18]. Thus creating a system where the neighbouring nitrogen lowered the working pH of the boronic acid and provided a fluorescence signalling mechanism to report saccharide binding (Figure 4).

**Figure 5:**
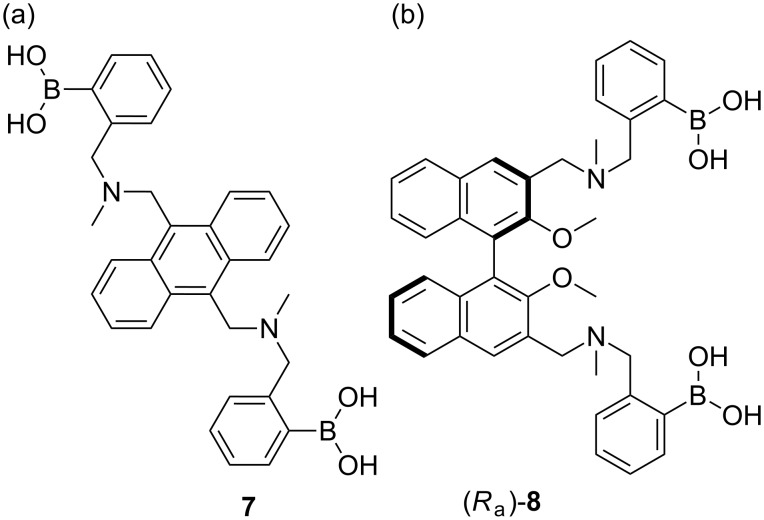
(a) Glucose selective PET system. (b) Chiral discriminating PET system.

Sensei (Seiji Shinkai) then gave me my next and most important academic lesson – if you discover “gold” in one area of research you should keep on digging in that area – the hope is that you will discover a “seam of gold” leading to even more research achievements. This result, as far as my career goes was a Eureka moment, for which I count myself very lucky, this very simple discovery was the start of a very big “seam of gold” that has continued to fuel my research to the present day [19–27]. After, we discovered this simple system we very quickly went on develop a glucose selective system [28–29] (Figure 5a). It is very rewarding to see that the basic framework of this glucose selective system is still be used by Takeuchi for the development of implantable sensors [30–32].

Some moments during your research career can be very memorable – one such moment was being confronted by Shinkai Sensei with a bottle of champagne to celebrate that our chiral discriminating system had just been accepted for publication in Nature [33] (Figure 5b).

At the 20th International Symposium on Macrocyclic Chemistry [34] in 1995, it was my great pleasure to meet and chat with Prof. J. Fraser Stoddart who had inspired me as PhD student to do research in the first place. He had recently moved to the University of Birmingham and suggested that I apply for a Royal Society University Research Fellowship to move and start my independent academic career in Birmingham.

I was very excited with the prospect of this opportunity and after a simple application procedure was very lucky to be awarded a Fellowship and so in 1996 I moved to Birmingham start my independent career. Before, I left Japan Sensei (Seiji Shinkai) gave me some excellent research advice by reminding me that – “even monkeys fall from trees” – in other words to remember as I moved to the next stage of my academic career, that everyone makes mistakes [35]. The time I spent in Japan was a very important time in my career and also life. Since, I left Japan with not only some excellent research results, more importantly I left with my Fiancée – Eriko Furukawa (we were married at St Michael's Church in Madeley on 3^rd^ May 1996).

The excellent research links I nurtured during my time at the Shinkai Chemirecognics project (1991–1995) have continued to flourish and in recognition of these collaborative research projects with Japan, myself and Seiji Shinkai as team leaders were awarded a Daiwa-Adrian Prize in 2013 [36] for “Chemonostics: Using chemical receptors in the development of simple diagnostic devices for age-related diseases” with a team including Steven Bull (University of Bath), John Fossey (University of Birmingham), Kazuo Sakurai [37–41] (University of Kitakyushu) and Yuji Kubo [42–47] (Tokyo Metropolitan University).

#### Independent research – Birmingham

**Figure 6:**
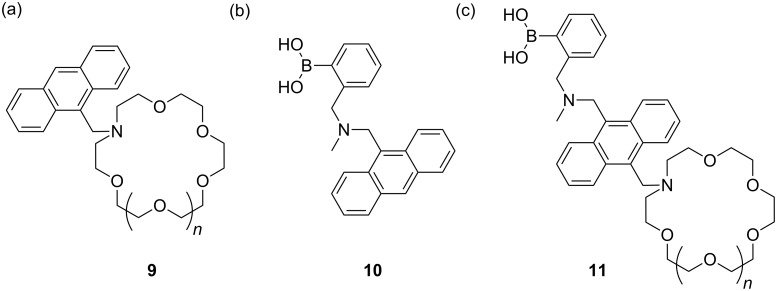
(a) Fluorescence photoinduced electron transfer (PET) cation sensors developed by A. P. De Silva. (b) Fluorescence photoinduced electron transfer (PET) saccharide sensor. (c) Fluorescence AND logic sensors for D-glucosamine hydrochloride.

Once again I was inspired by De Silva who gave a wonderful talk in Birmingham where he discussed fluorescence based logic systems [48]. During his talk I realised that you could easily combine the De Silva cation sensor [49] with the simple boronic acid sensor [18] to prepare a new logic based system. Therefore, with my first PhD student Chris Cooper [50] we combined these two receptor elements and developed an AND logic system for “cations” and “diols” (Figure 6) [51–52].

In 1998 Michael Bell from Beckman Coulter approached me about working together on a fluorescent glucose sensor system. Therefore, in July and few weeks after the birth of my son Joseph “Joey” Hiro Furukawa James, I visited Beckman-Coulter Inc., Advanced Technology Center in Brea, Orange County, California to discuss a project directed towards the development of fluorescent glucose sensors. Research towards modular systems was started in Birmingham with Susumu Arimori an excellent PDRF from the Shinkai group [53].

My time at the University of Birmingham had been rewarding, however, with a young family I felt that the security offered by a more permanent position was needed. As chance would have it at that time the University of Bath was looking for an Organic Chemistry Lecturer and in 2000 I moved to take a position in Bath. This was a great move for me from a personal as well as career perspective, since Bath was a very supportive and friendly department. I was also lucky to be appointed at the same time as Toby Jenkins and Steven Bull who have been very good friends and collaborators over the last 15 years.

#### Independent research – Bath

One of the first things I did at Bath was to expand and develop the modular saccharide selective fluorescent sensors which was part of the project with Beckmann-coulter started as a Royal Society University Research Fellow at the University of Birmingham [54–55]. My family also expanded on arriving in Bath with the birth of my daughter Elinor “Ellie” Yoko Furukawa James.

**Figure 7:**
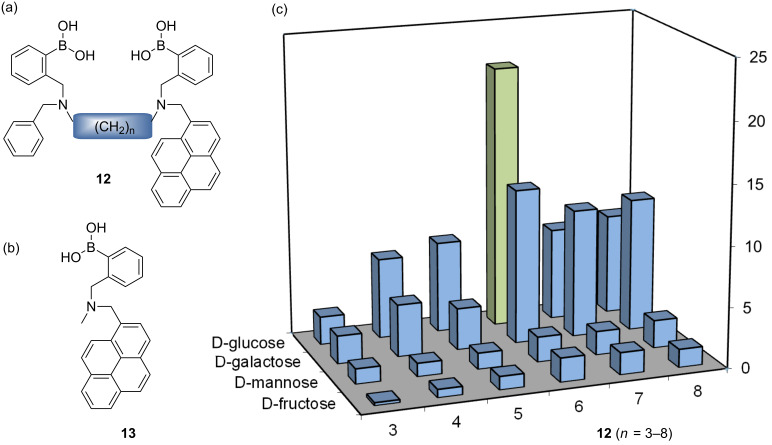
(a) Pyrene diboronic acids (*n* = 3–8). (b) Pyrene monoboronic acid. (c) Block chart showing the relative stability *K_rel_* of saccharide complexes. Obtained from the observed stability constants (*K*_obs_) for D-glucose, D-galactose, D-fructose and D-mannose with pyrene diboronic acids (*n* = 3–8) divided by the observed stability constants (*K*_obs_) with pyrene monoboronic acid, to yield relative values with saccharides*.*

**Figure 8:**
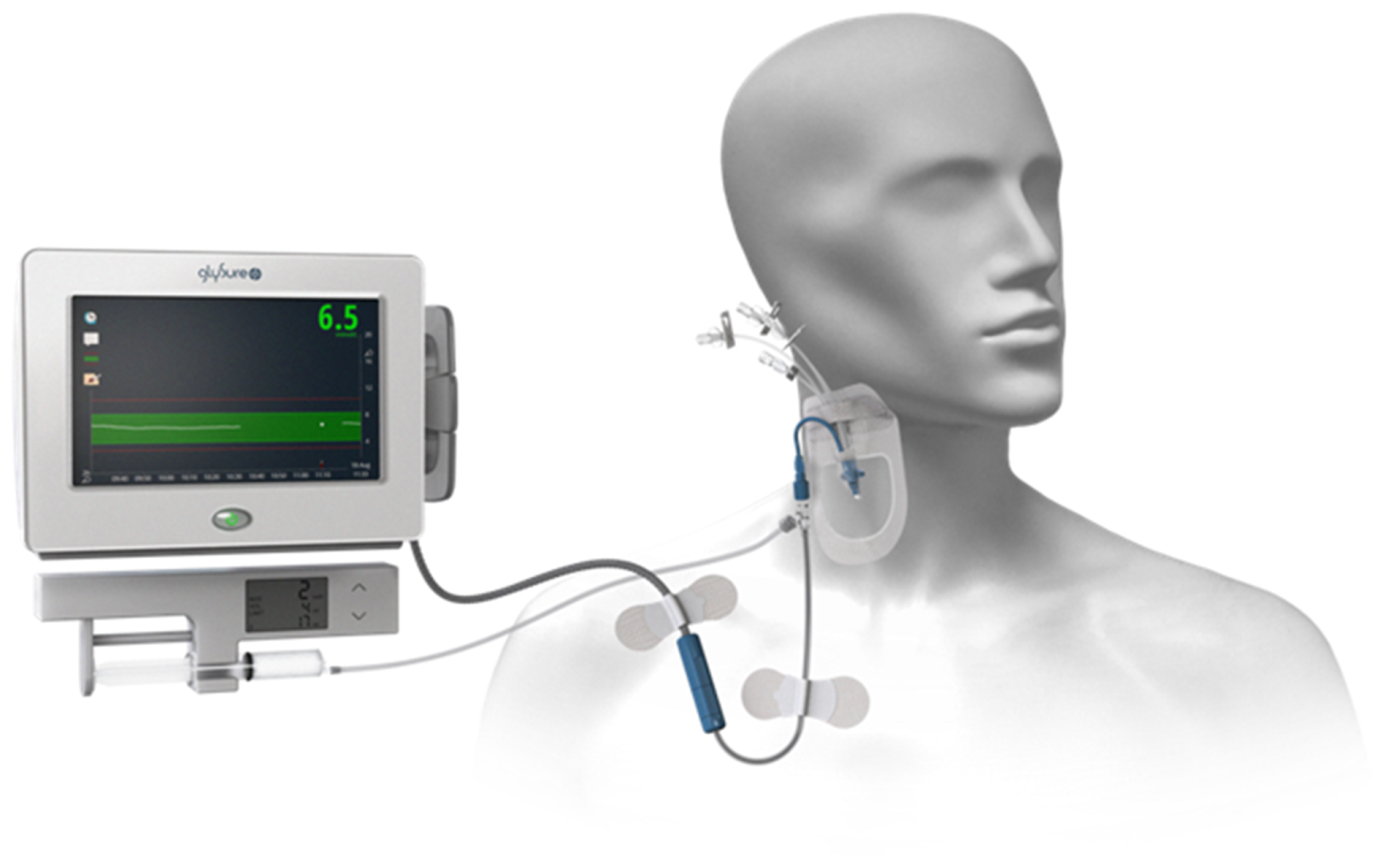
Glysure Continuous Intravascular Glucose Monitoring (CIGM) System. Image provided by Nicholas P. Barwell Glysure Ltd. [56].

As with many industrial projects Beckman-Coulter Inc. has subsequently moved on to other areas of research. However, as luck would happen Glysure Ltd. [56] in the form of Barry Crane approached me in 2006 about our fluorescent glucose selective systems, after reading our paper in *Perkin Transactions 1* [55], therefore, together we set about improving these systems in order to develop a practical sensor system for use in intensive care units (ICU) [57] (Figure 8). The collaboration with Glysure Ltd. continues to flourish and over the years has been funded by many routes including a TSB project [58] on “A Calibration Free Continuous Invasive Sensor Targeted at Glycaemic Control in the ICU”. This project is worthy of mention since it started a long standing association between myself and John S. Fossey who was the researcher co-investigator on the project.

The GlySure Continuous Intravascular Glucose Monitoring (CIGM) depicted in Figure 8 has recently secured a CE Mark as the world’s first and only Continuous Intravascular Glucose Monitoring System (CIGMS). The award of this CE Mark allows GlySure to market their device in Europe. They are currently conducting a UK-based trial to facilitate use of the device across all adult Intensive Care patients.

**Figure 9:**
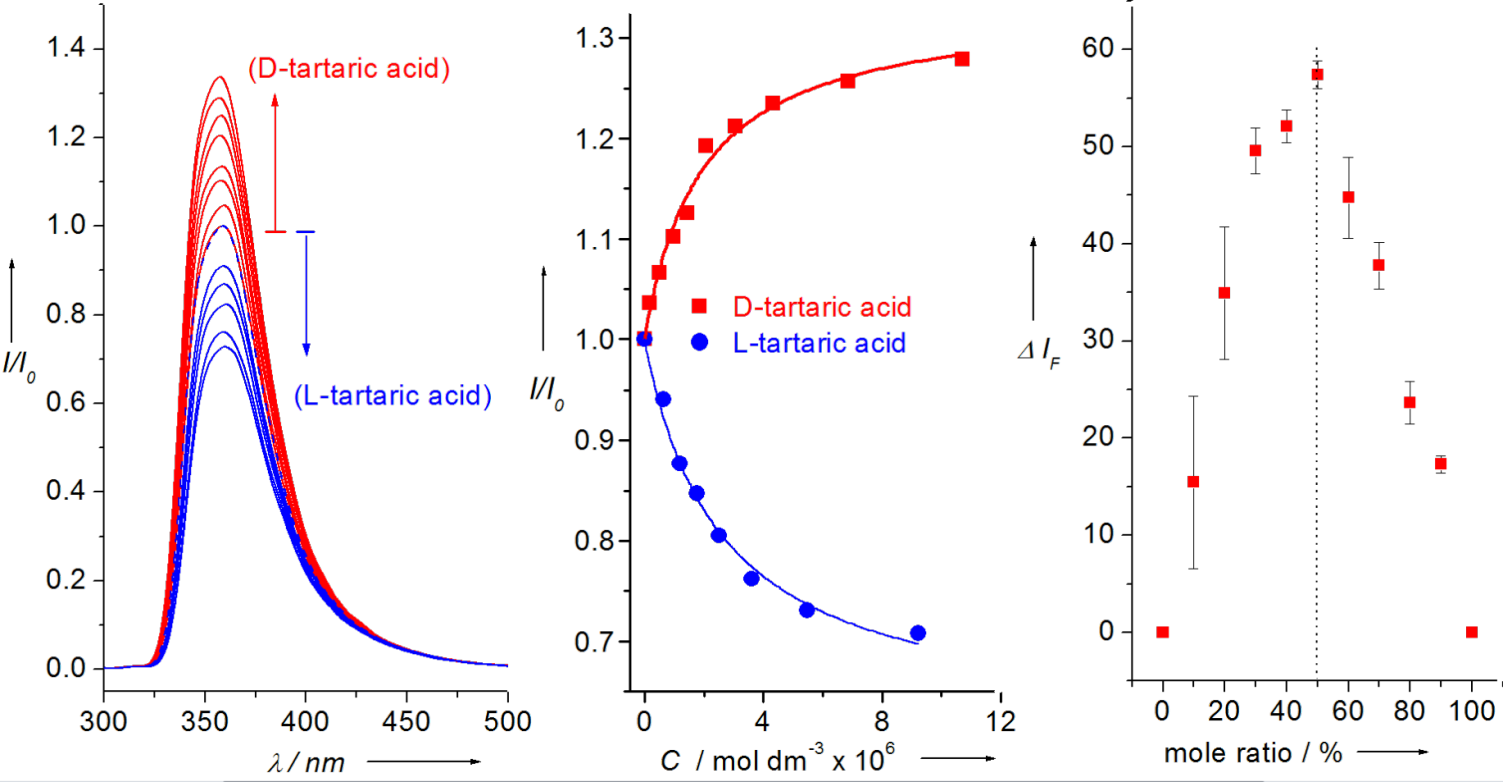
Chiral discrimination of D- and L-tartaric acid by (*R*)-**8** at pH 5.6. [(*R*)-**8**] = 5.0 × 10^−6^ mol dm^−3^, at pH 5.6 in 0.05 mol dm^−3^ NaCl (52.1% methanol in water), λ_ex_ at 289 nm, 22 °C. The pH was kept at 5.6 with NaOH/HCl. (Left) Emission spectra. (Center) Normalized emission intensity) as a function of added tartaric acid concentration. Lines are fit to 1:1 binding isotherm (Right) Job plot of (*R*)-**8** with D-tartaric acid at a constant total concentration [D-tartaric acid] + [*R*] = 5.0 × 10^−6^ mol dm^−3^; λ_em_ at 358 nm. Reproduced with permission from [61]. Copyright 2004 John Wiley and Sons.

Westheimer’s Discovery [59] – that “A couple of months in the laboratory can frequently save a couple of hours in the library” makes you realise how important it is to read and keep up to date with the literature. Taking that one step further, it is also very important to keep abreast of papers that cite your own work. This is clearly illustrated using a paper by Todd A. Houston, who discovered that the racemic form of our chiral binol boronic acid **8** formed very strongly complexes with tartaric acid [60]. This paper made us realise that our chiral binol boronic acid **8** should be able to discriminate the enantiomers of tartaric acid and also bind strongly with other sugar acids. Therefore, with Jianzhang Zhao an outstanding Postdoctoral Research Fellow in my group and currently a Professor at Dalian University of Technology, we decided to investigate the properties of the enantiomerically pure forms of the receptor with chiral tartaric acid and other sugar acids. It turned out that the chiral system **8** (*R* or *S*) was very good at differentiating the enantiomers of tartaric acid and also bound bind strongly with other sugar acids [61] (Figure 9).

**Figure 10:**
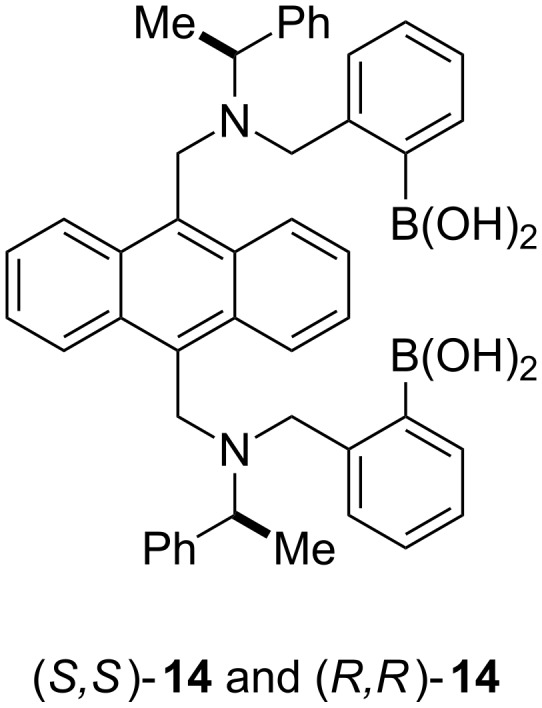
Chiral discriminating sensor (relative stereochemistry shown) constructed using a good fluorophore (anthracene).

While, we were very happy with these results, we realised that the system could be improved by changing the fluorophore from binol, to a much better fluorophore such as anthracene. Therefore, we combined the structural design of the glucose selective sensor **7** with chiral building blocks to prepare two sensor (*S*,*S*)-**14** and (*R*,*R*)-**14** (Figure 10).

**Figure 11:**
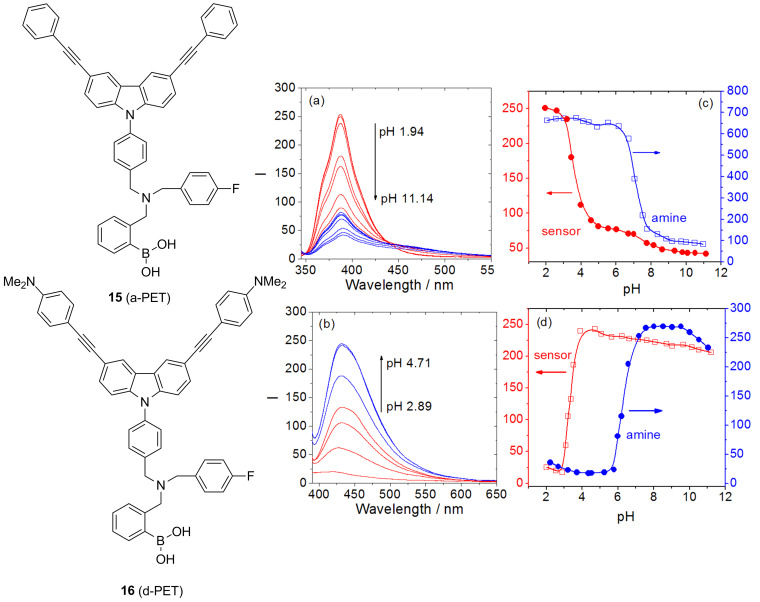
Fluorescence emission intensity-pH profile of: (a) Sensor **15**: 1.0 × 10^−6^ mol dm^−3^ (λ_ex_ 370 nm, λ_em_ 435 nm. (b) Sensor **16**: 1.0 × 10^−6^ mol dm^−3^ (λ_ex_ 335 nm, λ_em_ 390 nm). In 5.0 × 10^−2^ mol dm^−3^ NaCl ionic buffer (52.1% methanol in water). Emission spectra of the sensors (c) **15** (1.0 × 10^−6^ mol dm^−3^), λ_ex_ 370 nm; (d) **16** (1.0 × 10^−6^ mol dm^−3^), λ_ex_ 335 nm. Sensors in 5.0 × 10^−2^ mol dm^−3^ NaCl ionic buffer (52.1% methanol in water, w/w), 25 °C. Reproduced with permission from [64]. Copyright 2010 American Chemical Society.

After finishing as a Postdoctoral Research Fellow at Bath in 2005 Jianzhang Zhao took up an academic position at Dalian University of Technology. We have continued to collaborate and in particular we worked together to improve the chiral discriminating systems. In order to improve the chiral systems we designed sensors using a d-PET rather than the normal a-PET fluorescence sensing mechanism. With d-PET systems the fluorophore is the electron donor and the protonated amine/boronic acid moiety as the acceptor (the reverse is true for normal a-PET systems). Therefore, d-PET systems have a significant advantage over the a-PET systems at acidic pH, since background emission for these sensors is much lower. This is particularly important given that we were developing receptors for acidic guests such as tartaric acid and sugar acids [62–65] (Figure 11).

**Figure 12:**
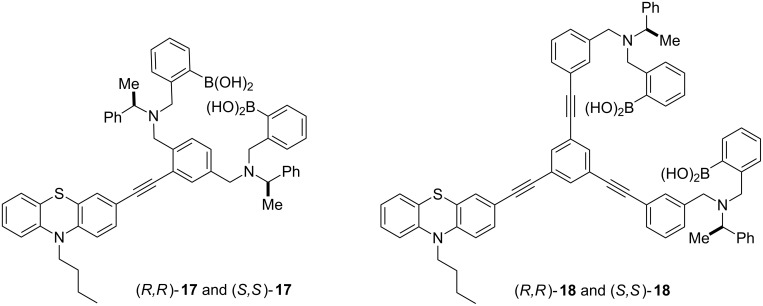
Modular chiral discriminating d-PET systems (relative stereochemistry shown).

The best chiral discriminating d-PET system was constructed using a phenothiazine fluorophore **17** and **18** [66]. The phenothiazine fluorophore was chosen because it is a very strong electron donor. These sensors resulted in an eight fold contrast ratio, which was much better than the two fold obtained for the carbazole-based d-PET fluorescent sensors. Using sensor **18** with the largest spacing between the boronic acid receptors resulted in excellent enantioselective discrimination of D- and L-tartaric acid (Figure 12).

**Figure 13:**
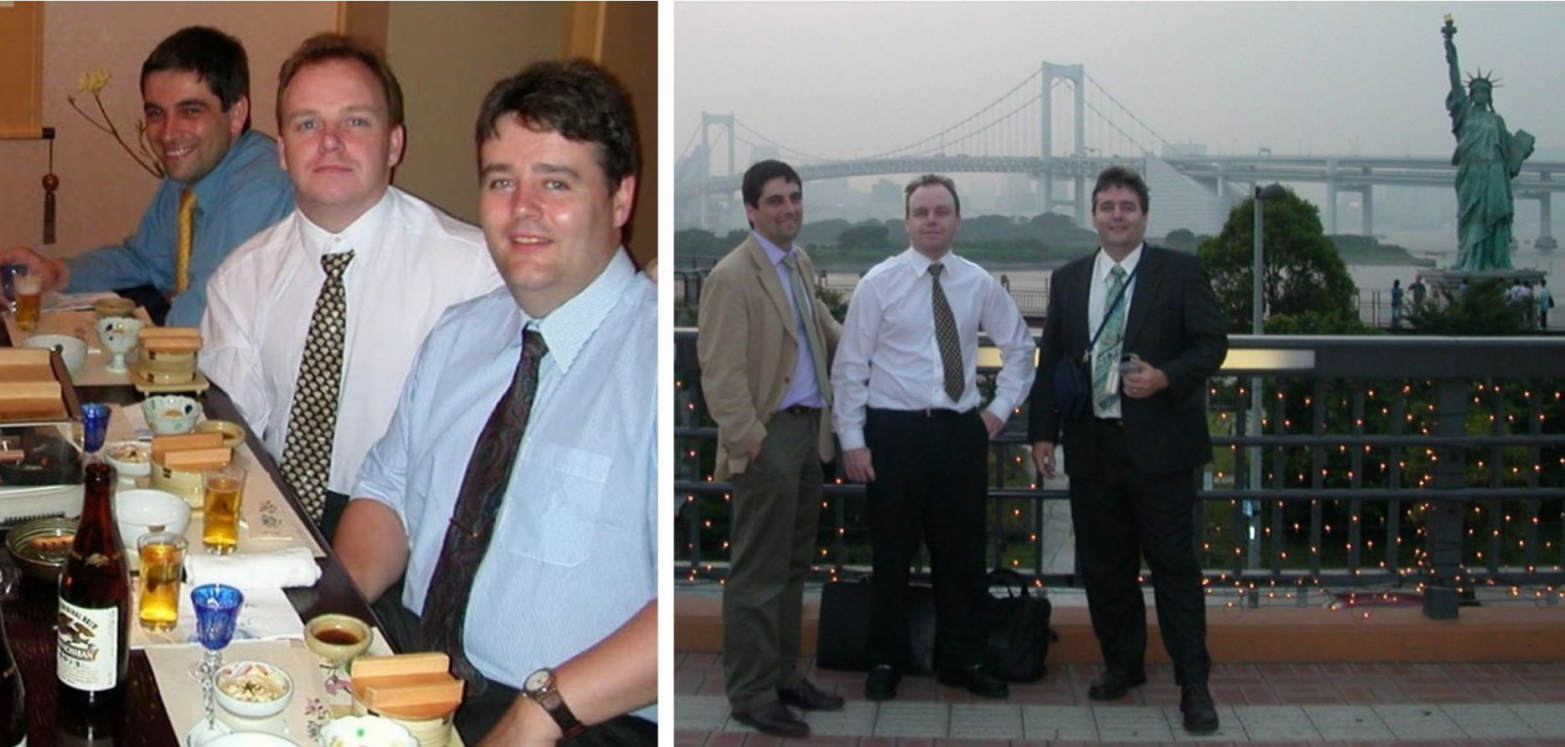
With Matthew Davidson and Steven Bull during “World Cup” lecture tour of Japan in 2002. (Left) Private photo taken in Osaka. (Right) Photo taken in Tokyo by Katsuhiko Ariga.

**Figure 14:**
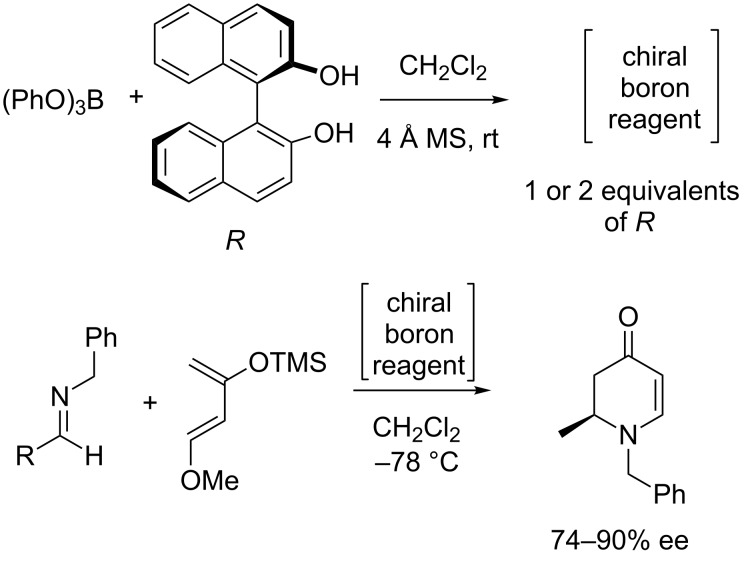
Preparation of chiral boron reagent and use as catalyst for aza-Diels–Alder reactions.

The Japan World Cup in 2002 was responsible for starting a number of enduring collaborations. Matthew Davidson, Steven Bull and myself spent approximately two weeks from May to June travelling around Japan presenting research talks at 13 research institutes and universities [67]. Two of the collaborations established during this trip are worthy of note since they became part of the team awarded the 2013 Daiwa-Adrian Prize. Yuji Kubo (TMU) who was at that time based at Saitama University, who I had first met during the XVIII ISMC in 1993 was one and the other was Kazuo Sakurai (Kitakyushu University) who as it happens had employed Susumu Arimori after he left my group at the end of 2001 as a Post-Doctoral Research Fellow. The two weeks on the road also allowed Steve and me to discuss research. Steve is interested in chiral catalysis and I am interested in chiral sensing so a collaborative project using boron as a chiral catalyst was a good idea. We quickly came up with a research plan over a beer or two (Figure 13). Then on our return to the UK we quickly put these ideas into practice with the investigation of a “chiral boron reagent” formed between binol and trimethoxy borate for the Lewis acid catalyst of diastereoselective aza-Diels–Alder reactions [68] (Figure 14). While, the structure of the “chiral boron reagent” still remains unknown during our investigation of analogues we discovered a very interesting three-component self-assembly.

**Figure 15:**
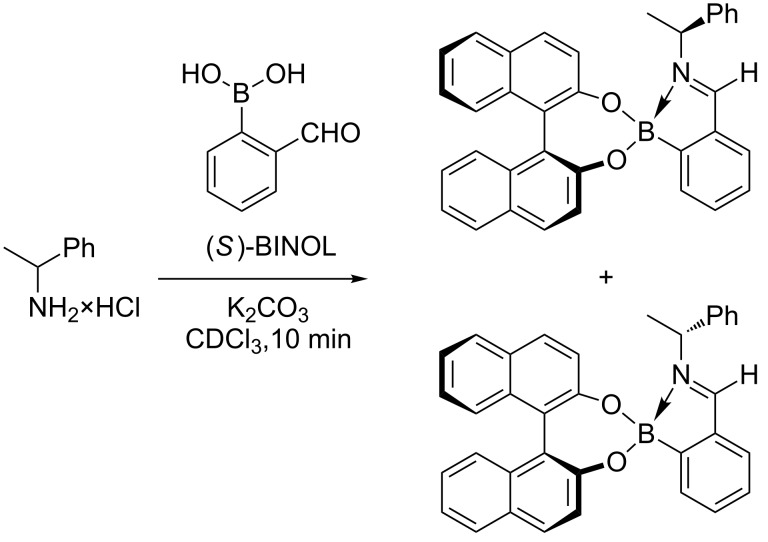
Chiral three component self-assembling system.

Chiral binol, a chiral amine and 2-formylbenzeneboronic acid spontaneously self-assemble to quantitatively form a stable complex. Interestingly, when the chirality of the binol or amine was fixed the ^1^H NMR of the complexes formed using a scalemic mixture of the other chiral partner (diol or amine) results in a ^1^H NMR where signals due to both diastereomeric complexes are clearly separated. The diastereomeric imine signals are particularly useful since they are in a region of the spectra clear of interference from signals due to the binol (diol) or amine. Integration of the pair of imine signals provides a diastereomeric ratio which can be related to the enantiomeric excess of the original scalemic mixture of binol (diol) or amine (Figure 15).

The three-component system was very versatile and we could use the complexes to determine the enantiomeric excess (ee) of amines [69–70], diamines [71], amino alcohols [72], hydroxylamines [73] and diols [74–75] using predominantly ^1^H NMR but we also demonstrated that by using 4-fluoro-2-formylbenzeneboronic acids then ^19^F NMR could also be used [76]. We have also used the three component systems in collaboration with Jim Tucker (University of Birmingham) to measure the ee of binol via electrochemistry [77]. The system has also been used with Eric Anslyn (University of Texas at Austin) to determine the ee of amines using circular dichroism (CD) spectroscopy [78]. The system was more recently used to measure the ee of amines using fluorescence [79].

The UK Office of Science and Innovation (OSI) signed a bilateral agreement with the Chinese Ministry of Education in April 2007, making funding available for the scheme to develop enduring collaborations between UK and Chinese scientists. The UK and China had clearly recognised the need to bring together scientists to develop new networks. The programme was looked after by the Royal Society in the UK and and the China Scholarship Council (CSC) in China.

This was great timing given my good links with Jianzhang Zhao and having just returned from East China University of Science and Technology (ECUST) in Shanghai where I presented at the International Conference on Molecular Machines and Sensor (ICMMS). Therefore, we Jianzhang Zhao and Tony James (with Steven Bull and John Fossey) applied for funding to host a Thematic Workshop during 2008 on “Catalysis and Sensing for our Environment” (CASE) in Bath. We were very lucky in obtaining the funding [80] and the CASE Network was established [81–83].

*“A scientist has to work very hard to get to the point where he can be lucky.”* – Robert B. Woodward (1917–1979)

The 7^th^ Catalysis and Sensing for our Environment Symposium was held in Dublin on 9–10 July 2015 and jointly hosted in Ireland by Thorfinnur Gunnlaugsson (Trinity College Dublin), Donal O’Shea (Royal College of Surgeons in Ireland) and Robert Elmes (Maynooth University) [84]. It was my great honour to be presented an Inaugural CASE prize by Eric Anslyn at this meeting for my contributions to sensing and helping establish the CASE Network [85].

Thanks to the CASE Network enduring collaborations have been established in China with outstanding academics including Yun-Bao Jiang [86–93], Xuhong Qian [94–96], Yitao Long [97–106], Weiping Deng [107] ,Weihong Zhu [108–113] and Xiao-Peng He (Franck) [114–118]. In addition excellent, links and collaborations with other countries have also developed as a direct result of these networking meetings. Including the recent collaboration on chiral discrimination with Pavel Anzenbacher, Jr. (Bowling Green State University) and Tsuyoshi Minami (Yamagata University) [79,119] that was originated during discussion over several Margarita’s at CASE2013, which was hosted by Eric Anslyn and Jonathan Sessler in the University of Texas at Austin.

The ethos of the CASE meetings since the onset has been to bring together world leading researchers and junior scientists on an equal footing and to provide an environment where research ideas can be openly discussed in order to establish new collaborations and develop new ideas.

### The future

*“Prediction is very difficult, especially if it's about the future.”* – Niels Bohr (1885–1962)

The problem with predicting the future of research is that as scientists we are very literal and specific, but, it is much easier to be correct if your predictions are broad based. Consider the book “*Les Propheties*” by Nostradamus, while his predictions may be seen as vague, this makes them open to different interpretations and as such they are as topical and up-to-date as when they were written.

*“Study the past, if you would divine the future.”* – Confucius (551–479 BC)

This quote is close to my heart – you will see from this perspective that many of the advances in research that I have been involved with have to some extent required an understanding of the past. This is probably true for all scientists since the past is the published literature and we rely on these publications to build the future.

The outlook for boronic acid based receptors is very bright with many new areas of research on the horizon and many results near the cusp for translation into practical systems or devices in the near future. In particular Glysure Ltd. has demonstrated that boronic acid based receptors can be used to continuously monitor glucose concentrations in the ICU. The clear success of this system, points to the potential future use of this type of fluorescent boronic acid based glucose sensor for home use and the potential to incorporate the sensor into devices for closed loop treatment of diabetic patients.

One important practical application for boronic acid based receptors that is currently emerging is in the measurement of protein glycation. Glycated proteins are potentially important biomarkers for sugar-related non-communicable disease states, such as atherosclerosis, autoimmune diseases, cancer, and Alzheimer’s disease (AD). Some researchers have even coined the phrase Type 3 diabetes for AD. While it remains to be determined whether the formation of AGEs in AD, and other age-related diseases, is a primary or secondary event, detection of specific glycated proteins may provide a very useful tool for diagnosis, monitoring, and treatment of the increasingly wide range of disease states that are associated with uncontrolled levels of glucose in the bloodstream.

With Jean van den Elsen (University of Bath) we have developed a simple and cost-effective analytical technique for the detection of glycated proteins in a variety of biological samples, including plasma and brain homogenates. This electrophoresis-based analytical method evolved from our work with John Fossey [120] and relies on the reversible covalent binding of a fluorescent boronic acid to glycated proteins that enables them to be detected [121–123]. This fluorescent phenylboronic acid gel electrophoresis method (Flu-PAGE), is currently being used to analyse the glycated protein profiles of brain homogenates from an AD mouse model, human cortex homogenates and plasma and serum samples from diabetes sufferers, with the aim of identifying disease-specific glycated proteins.

The ultimate aim of this work is to develop early stage diagnostic tests allowing for faster intervention and treatment; in the future it may be possible to cure a condition before the patient is even aware of symptoms. Obviously these developments will also facilitate the evaluation of particular intervention and ultimately improve in the efficiency of treatment strategies.

The two examples given above, one for the home and closed loop treatment of diabetes and the other for a simple diagnostic test for Alzheimer’s disease (AD), clearly demonstrate that the near future of boronic acid based receptor research is assured. What about over the longer term? Rather than give specific predictions, I prefer to be more like Nostradamus and provide a prophecy, in which I predict that in the future the most important applications for boronic acid based receptors will be in the area of disease theranostics. In part these applications will develop as a direct result of the increased importance of animal, tissue and cellular imaging techniques using boronic acid receptors [124].

“*Education is the passport to the future, for tomorrow belongs to those who prepare for it today.*” – Malcolm X (1925–1965)

## Author biography


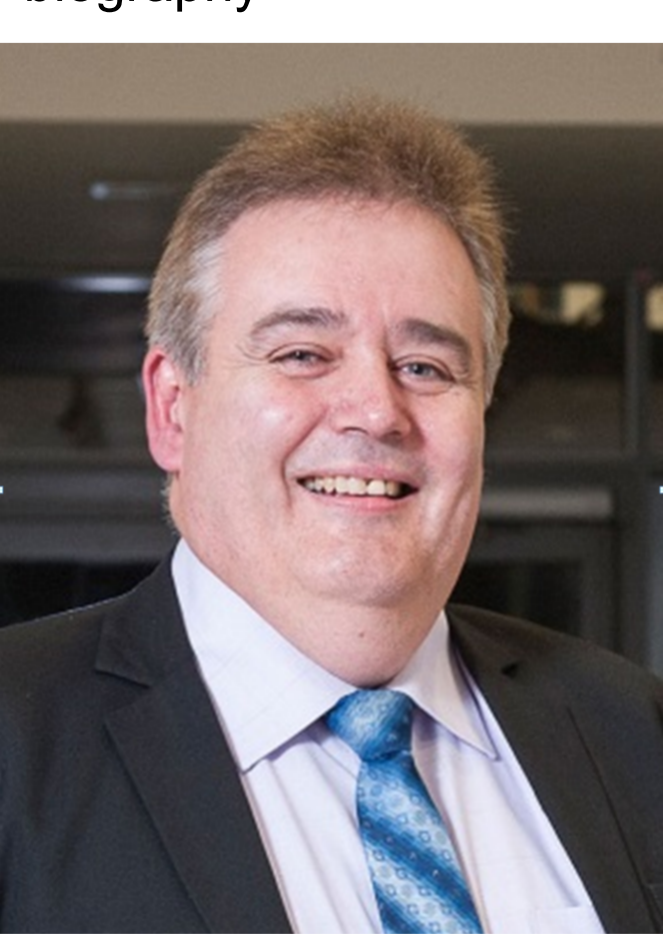


**Tony D. James** is a Professor at the University of Bath, he obtained his BSC from UEA (1986), PhD from the University of Victoria (1991) and was a PDRF with Seiji Shinkai in Japan (1991–1995). He was a Royal Society Research Fellow at the University of Birmingham (1995–2000), and University of Bath (2000–2003). He has been a visiting professor at Tsukuba, Osaka and Kyushu Universities, an AMADEus invited professor at the University of Bordeaux and is a guest Professor at East China University of Science and Technology, Xiamen University, Shandong Normal University, Nanjing University and is a Hai-Tian (Sea-Sky) Scholar at Dalian University of Technology. In 2013 he was awarded a Daiwa-Adrian Prize and in 2015 received the Inaugural Catalysis and Sensing for our Environment (CASE) Prize. His research interests include boronic acid based receptors for the fluorescence sensing of saccharides.
